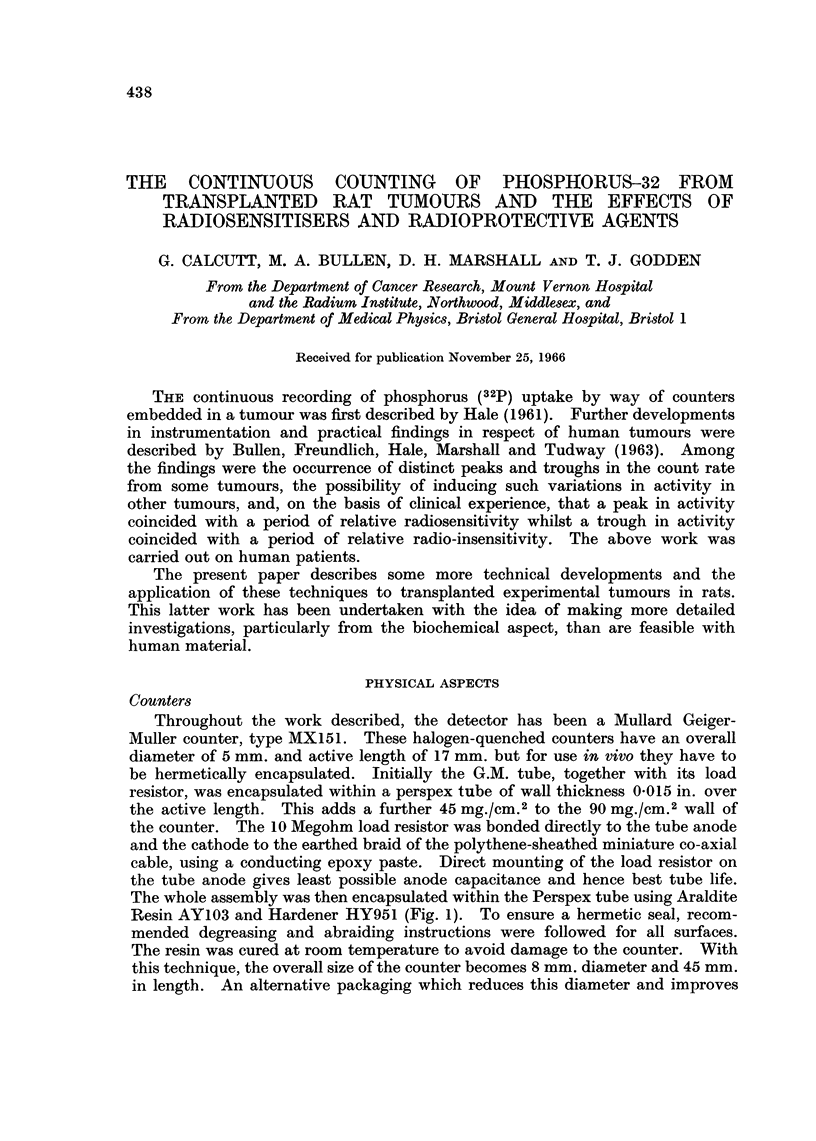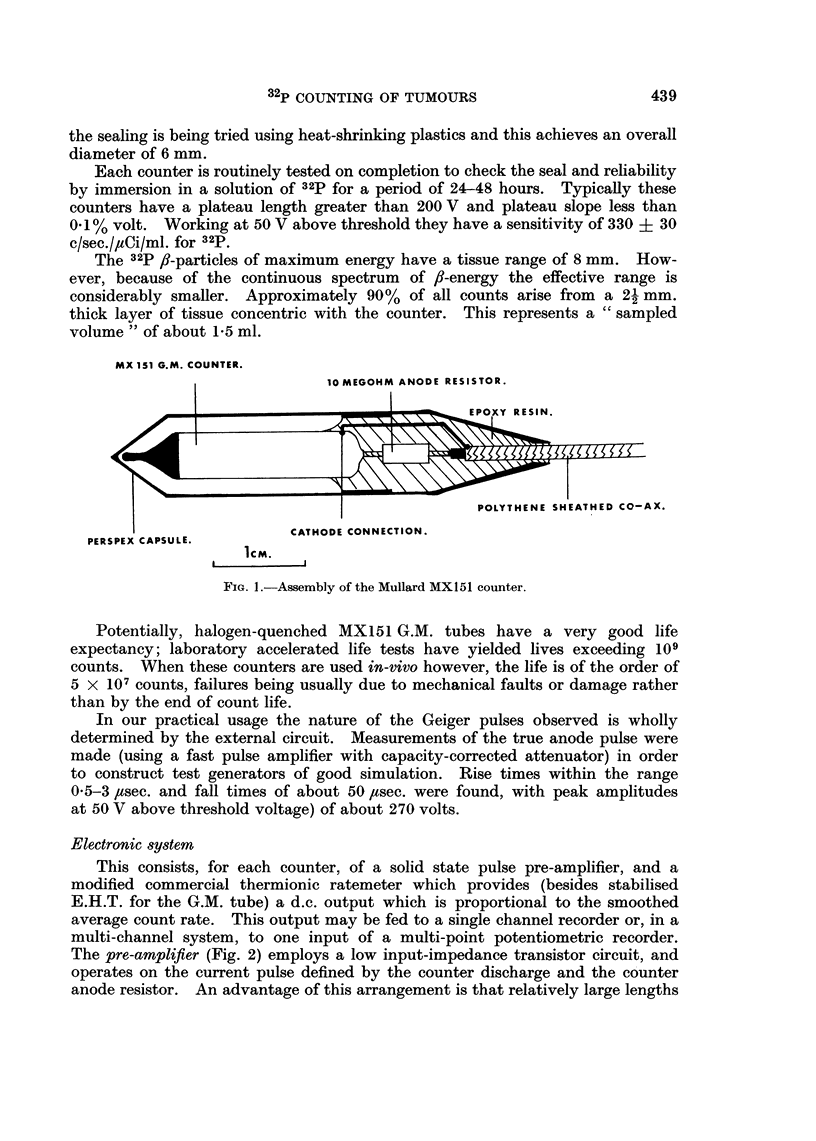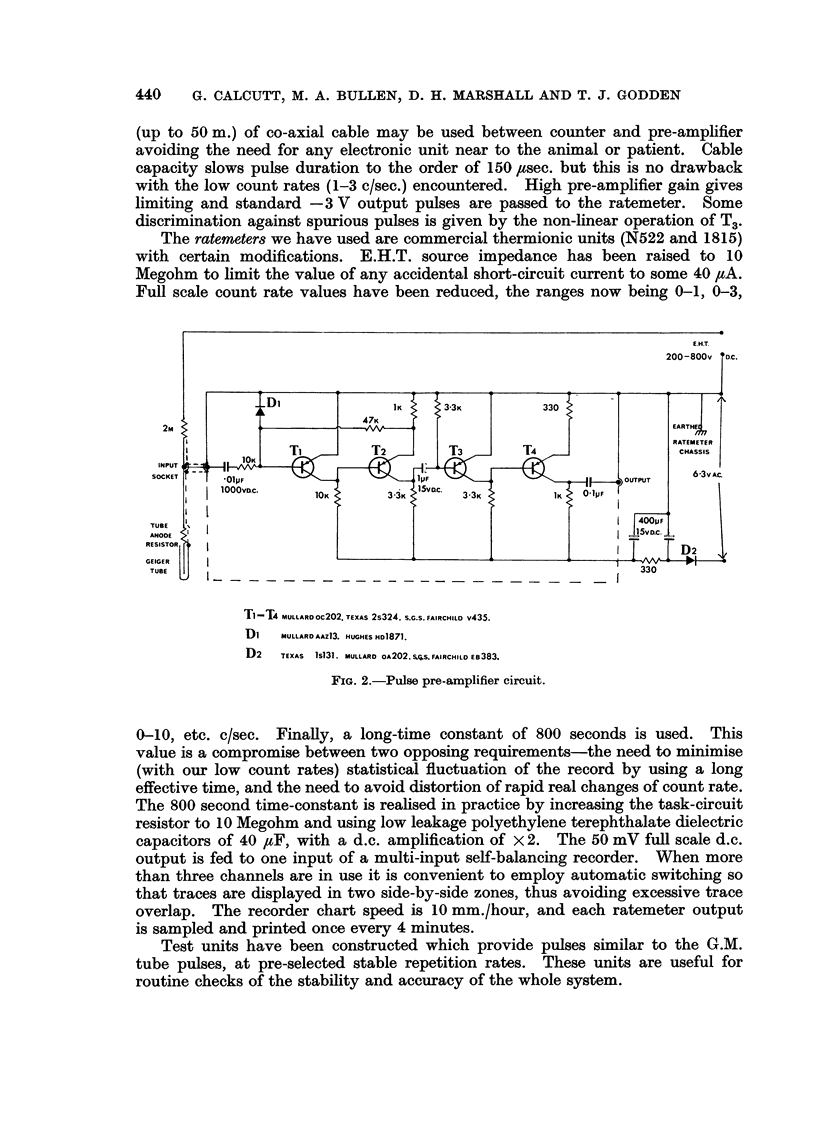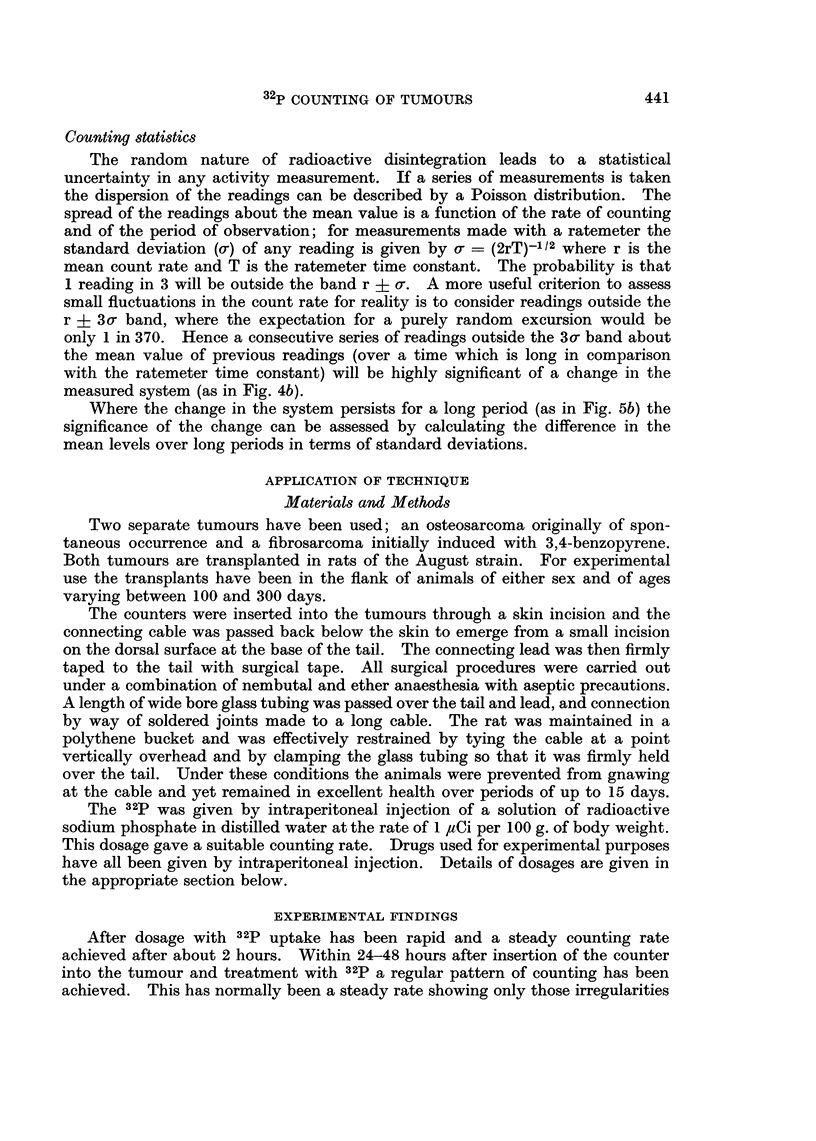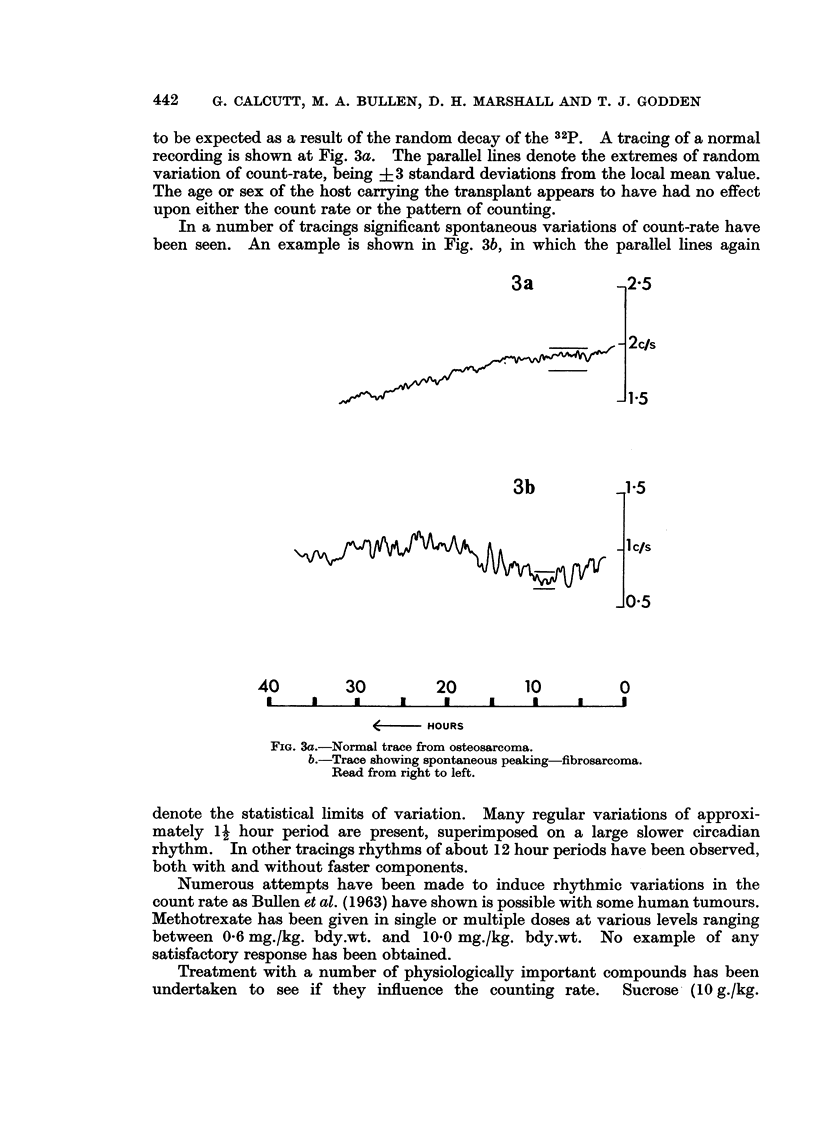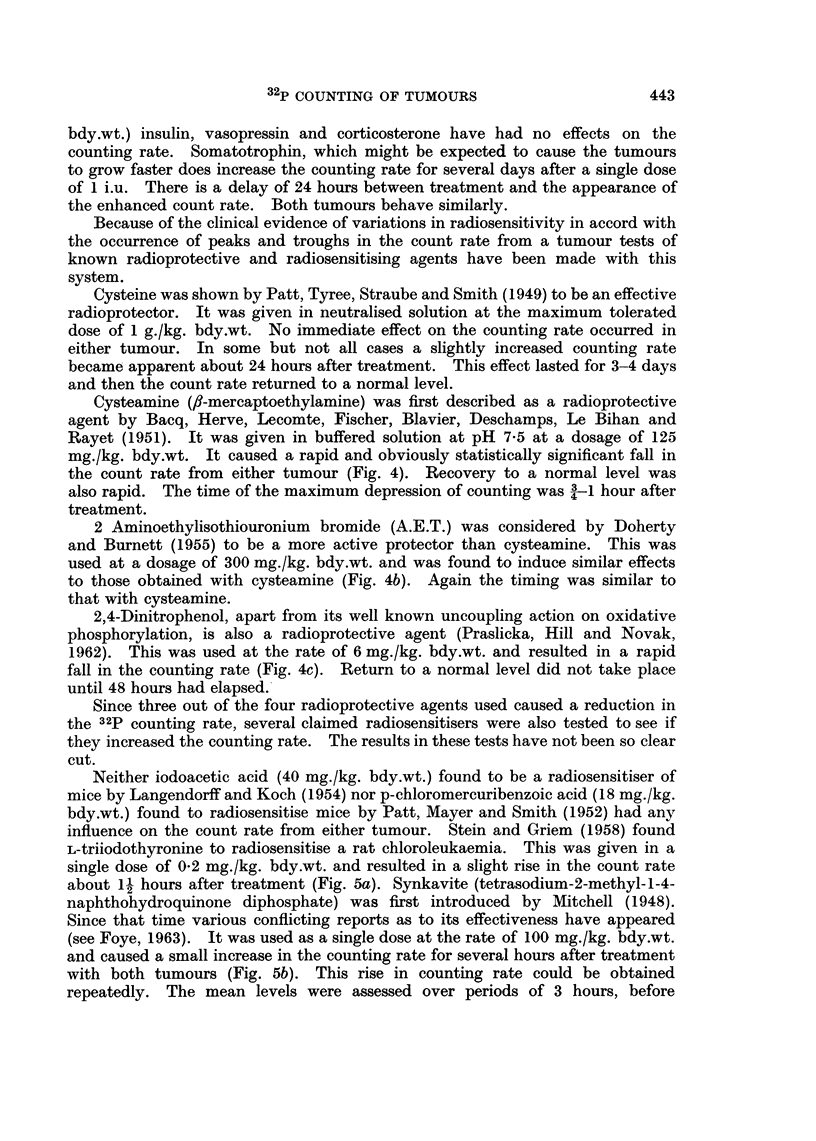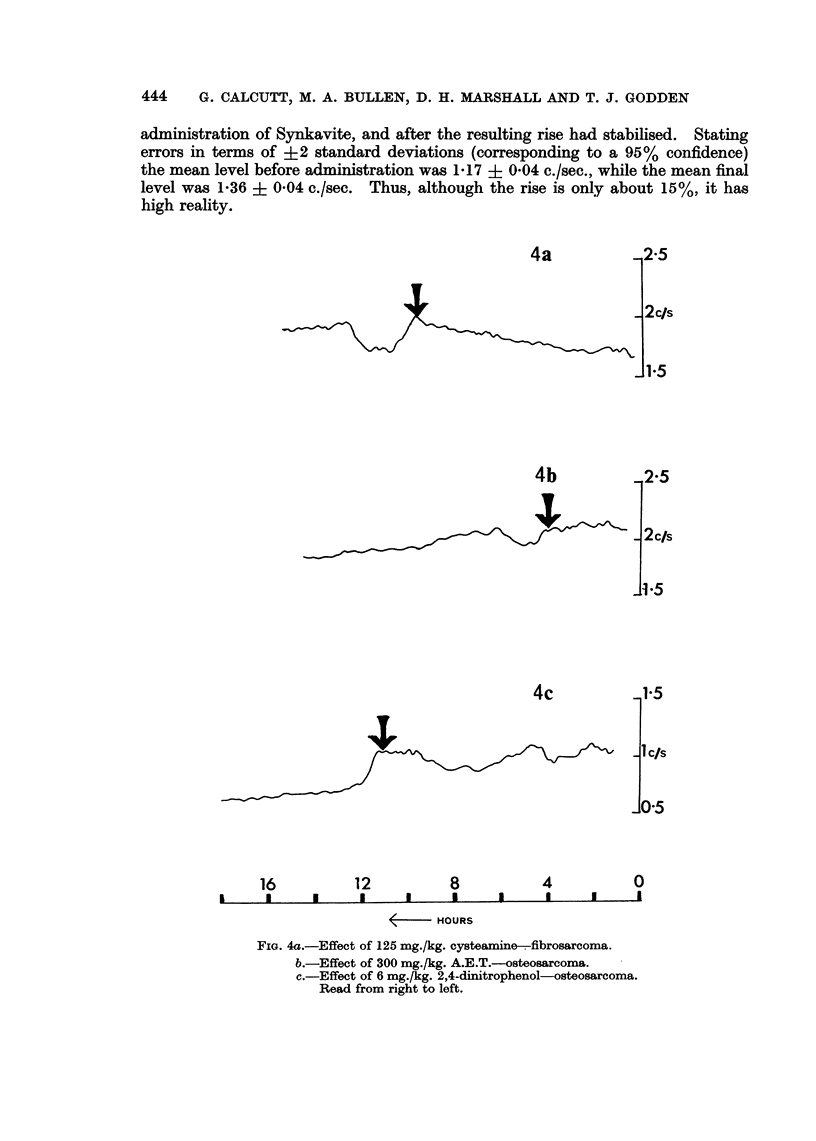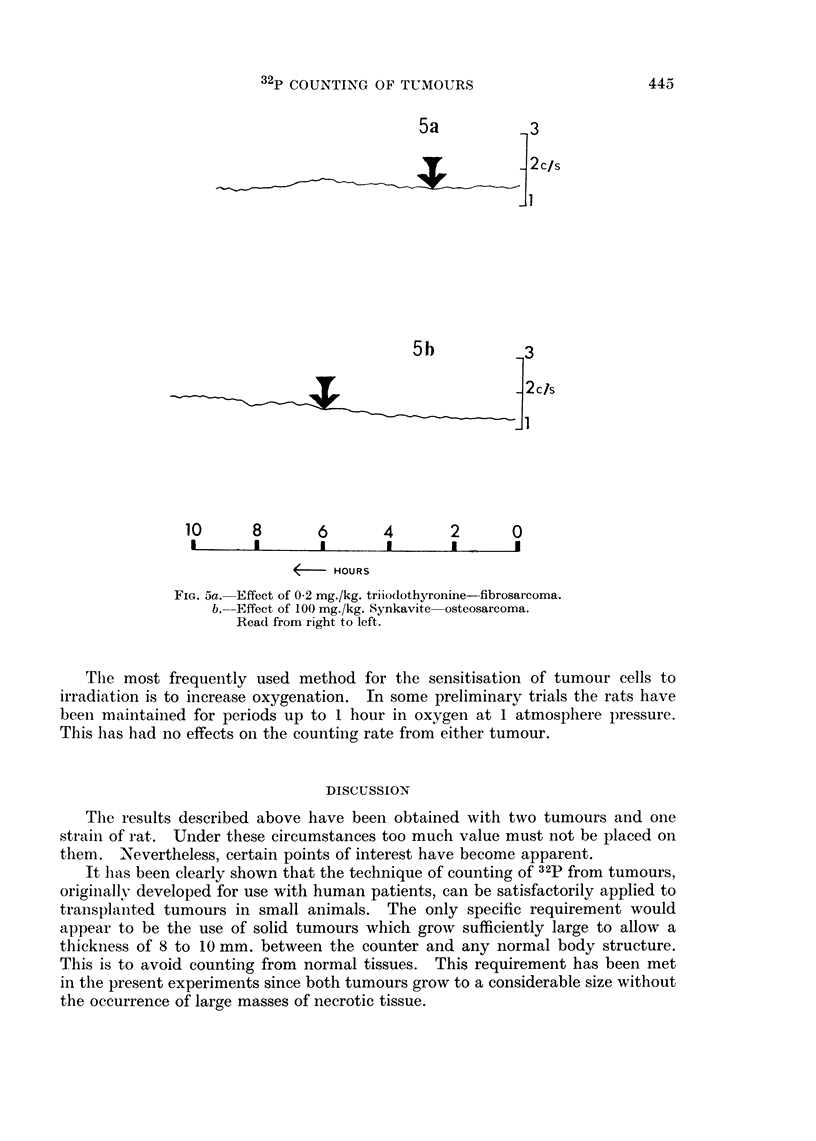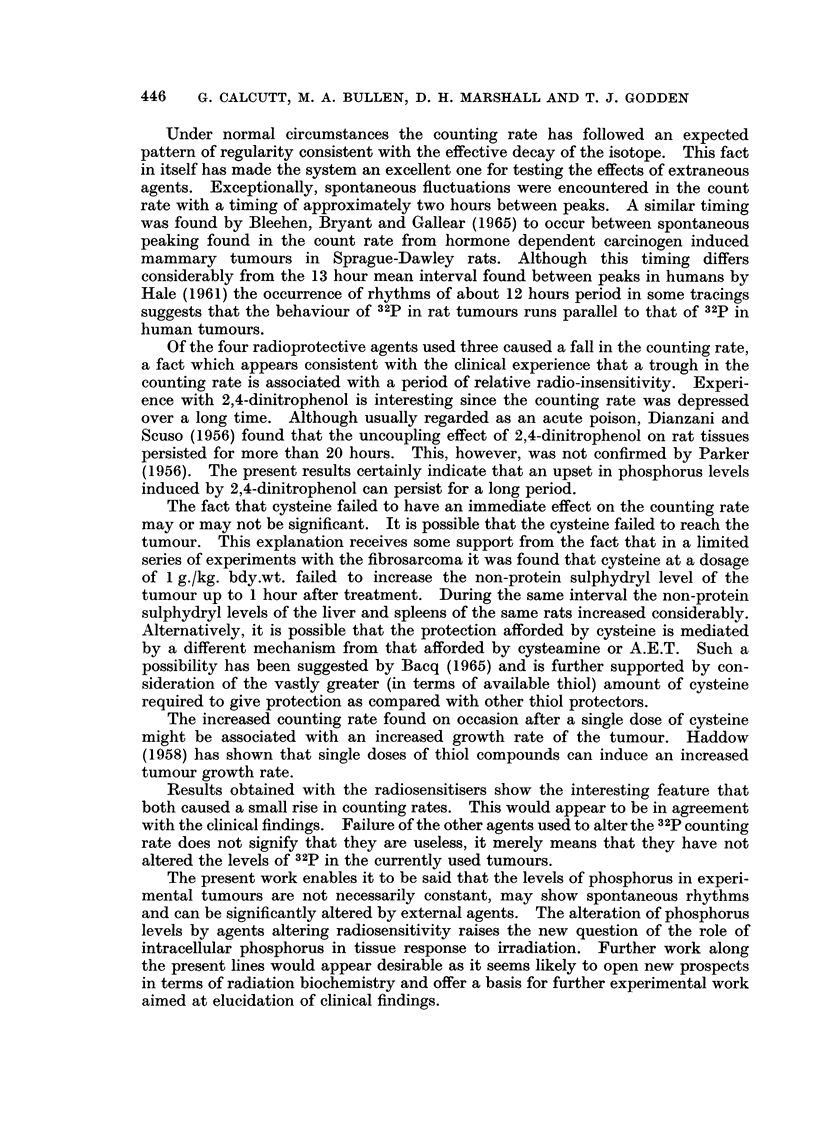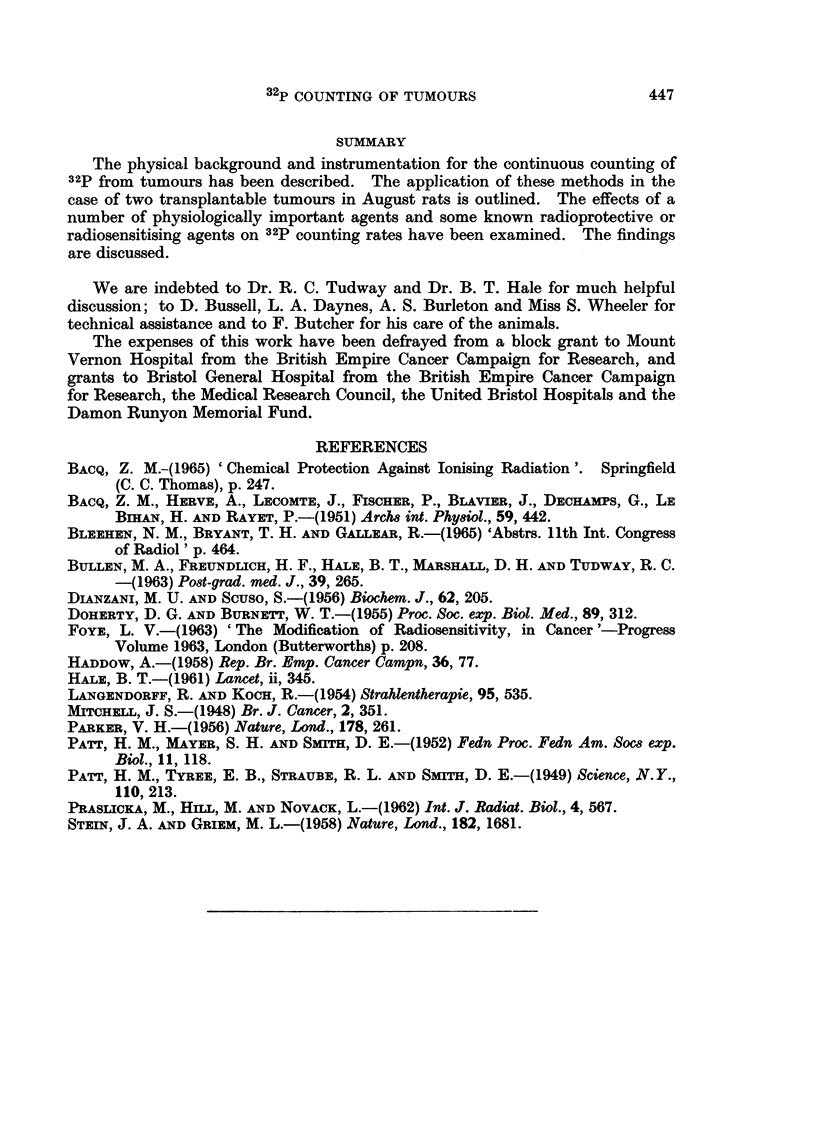# The continuous counting of phosphorus-32 from transplanted rat tumours and the effects of radiosensitisers and radioprotective agents.

**DOI:** 10.1038/bjc.1967.50

**Published:** 1967-06

**Authors:** G. Calcutt, M. A. Bullen, D. H. Marshall, T. J. Godden


### THE CONTINUOUS COUNTING OF PHOSPHORUS-32 FROM TRANSPLANTED RAT TUMOURS AND THE EFFECTS OF RADIOSENSITISERS AND RADIOPROTECTIVE AGENTS

G. CALCUTT, M. A. BULLEN, D. H. MARSHALL AND T. J. GODDEN

*From the Department of Cancer Research, Mount Vernon Hospital and the Radium Institute, Northwood, Middlesex, and From the Department of Medical Physics, Bristol General Hospital, Bristol* 1

Received for publication November 25, 1966

THE continuous recording of phosphorus ($^{32}P$) uptake by way of counters embedded in a tumour was first described by Hale (1961). Further developments in instrumentation and practical findings in respect of human tumours were described by Bullen, Freundlich, Hale, Marshall and Tudway (1963). Among the findings were the occurrence of distinct peaks and troughs in the count rate from some tumours, the possibility of inducing such variations in activity in other tumours, and, on the basis of clinical experience, that a peak in activity coincided with a period of relative radiosensitivity whilst a trough in activity coincided with a period of relative radio-insensitivity. The above work was carried out on human patients.

The present paper describes some more technical developments and the application of these techniques to transplanted experimental tumours in rats. This latter work has been undertaken with the idea of making more detailed investigations, particularly from the biochemical aspect, than are feasible with human material.

### PHYSICAL ASPECTS

### *Counters*

Throughout the work described, the detector has been a Mullard Geiger-Muller counter, type MX151. These halogen-quenched counters have an overall diameter of 5 mm. and active length of 17 mm. but for use *in vivo* they have to be hermetically encapsulated. Initially the G.M. tube, together with its load resistor, was encapsulated within a perspex tube of wall thickness 0·015 in. over the active length. This adds a further 45 mg./cm.$^2$ to the 90 mg./cm.$^2$ wall of the counter. The 10 Megohm load resistor was bonded directly to the tube anode and the cathode to the earthed braid of the polythene-sheathed miniature co-axial cable, using a conducting epoxy paste. Direct mounting of the load resistor on the tube anode gives least possible anode capacitance and hence best tube life. The whole assembly was then encapsulated within the Perspex tube using Araldite Resin AY103 and Hardener HY951 (Fig. 1). To ensure a hermetic seal, recommended degreasing and abraiding instructions were followed for all surfaces. The resin was cured at room temperature to avoid damage to the counter. With this technique, the overall size of the counter becomes 8 mm. diameter and 45 mm. in length. An alternative packaging which reduces this diameter and improves the sealing is being tried using heat-shrinking plastics and this achieves an overall diameter of 6 mm.

Each counter is routinely tested on completion to check the seal and reliability by immersion in a solution of $^{32}$P for a period of 24–48 hours. Typically these counters have a plateau length greater than 200 V and plateau slope less than 0·1% volt. Working at 50 V above threshold they have a sensitivity of 330 $\pm$ 30 c/sec./$\mu$Ci/ml. for $^{32}$P.

The $^{32}$P $\beta$-particles of maximum energy have a tissue range of 8 mm. However, because of the continuous spectrum of $\beta$-energy the effective range is considerably smaller. Approximately 90% of all counts arise from a $2\frac{1}{2}$ mm. thick layer of tissue concentric with the counter. This represents a " sampled volume " of about 1·5 ml.

FIG. 1.—Assembly of the Mullard MX151 counter.

Potentially, halogen-quenched MX151 G.M. tubes have a very good life expectancy; laboratory accelerated life tests have yielded lives exceeding $10^9$ counts. When these counters are used *in-vivo* however, the life is of the order of $5 \times 10^7$ counts, failures being usually due to mechanical faults or damage rather than by the end of count life.

In our practical usage the nature of the Geiger pulses observed is wholly determined by the external circuit. Measurements of the true anode pulse were made (using a fast pulse amplifier with capacity-corrected attenuator) in order to construct test generators of good simulation. Rise times within the range 0·5–3 $\mu$sec. and fall times of about 50 $\mu$sec. were found, with peak amplitudes at 50 V above threshold voltage) of about 270 volts.

*Electronic system*

This consists, for each counter, of a solid state pulse pre-amplifier, and a modified commercial thermionic ratemeter which provides (besides stabilised E.H.T. for the G.M. tube) a d.c. output which is proportional to the smoothed average count rate. This output may be fed to a single channel recorder or, in a multi-channel system, to one input of a multi-point potentiometric recorder. The *pre-amplifier* (Fig. 2) employs a low input-impedance transistor circuit, and operates on the current pulse defined by the counter discharge and the counter anode resistor. An advantage of this arrangement is that relatively large lengths

(up to 50 m.) of co-axial cable may be used between counter and pre-amplifier avoiding the need for any electronic unit near to the animal or patient. Cable capacity slows pulse duration to the order of 150 $\mu$sec. but this is no drawback with the low count rates (1–3 c/sec.) encountered. High pre-amplifier gain gives limiting and standard $-3$ V output pulses are passed to the ratemeter. Some discrimination against spurious pulses is given by the non-linear operation of $T_3$.

The *ratemeters* we have used are commercial thermionic units (N522 and 1815) with certain modifications. E.H.T. source impedance has been raised to 10 Megohm to limit the value of any accidental short-circuit current to some 40 $\mu$A. Full scale count rate values have been reduced, the ranges now being 0–1, 0–3,

T1–T4 MULLARD OC202. TEXAS 2S324. S.G.S. FAIRCHILD V435.

D1 MULLARD AAZ13. HUGHES HD1871.

D2 TEXAS 1S131. MULLARD OA202. S.G.S. FAIRCHILD EB383.

FIG. 2.—Pulse pre-amplifier circuit.

0–10, etc. c/sec. Finally, a long-time constant of 800 seconds is used. This value is a compromise between two opposing requirements—the need to minimise (with our low count rates) statistical fluctuation of the record by using a long effective time, and the need to avoid distortion of rapid real changes of count rate. The 800 second time-constant is realised in practice by increasing the task-circuit resistor to 10 Megohm and using low leakage polyethylene terephthalate dielectric capacitors of 40 $\mu$F, with a d.c. amplification of $\times 2$. The 50 mV full scale d.c. output is fed to one input of a multi-input self-balancing recorder. When more than three channels are in use it is convenient to employ automatic switching so that traces are displayed in two side-by-side zones, thus avoiding excessive trace overlap. The recorder chart speed is 10 mm./hour, and each ratemeter output is sampled and printed once every 4 minutes.

Test units have been constructed which provide pulses similar to the G.M. tube pulses, at pre-selected stable repetition rates. These units are useful for routine checks of the stability and accuracy of the whole system.

*Counting statistics*

The random nature of radioactive disintegration leads to a statistical uncertainty in any activity measurement. If a series of measurements is taken the dispersion of the readings can be described by a Poisson distribution. The spread of the readings about the mean value is a function of the rate of counting and of the period of observation; for measurements made with a ratemeter the standard deviation ($\sigma$) of any reading is given by $\sigma = (2rT)^{-1/2}$ where r is the mean count rate and T is the ratemeter time constant. The probability is that 1 reading in 3 will be outside the band $r \pm \sigma$. A more useful criterion to assess small fluctuations in the count rate for reality is to consider readings outside the $r \pm 3\sigma$ band, where the expectation for a purely random excursion would be only 1 in 370. Hence a consecutive series of readings outside the $3\sigma$ band about the mean value of previous readings (over a time which is long in comparison with the ratemeter time constant) will be highly significant of a change in the measured system (as in Fig. 4*b*).

Where the change in the system persists for a long period (as in Fig. 5*b*) the significance of the change can be assessed by calculating the difference in the mean levels over long periods in terms of standard deviations.

### APPLICATION OF TECHNIQUE

### *Materials and Methods*

Two separate tumours have been used; an osteosarcoma originally of spontaneous occurrence and a fibrosarcoma initially induced with 3,4-benzopyrene. Both tumours are transplanted in rats of the August strain. For experimental use the transplants have been in the flank of animals of either sex and of ages varying between 100 and 300 days.

The counters were inserted into the tumours through a skin incision and the connecting cable was passed back below the skin to emerge from a small incision on the dorsal surface at the base of the tail. The connecting lead was then firmly taped to the tail with surgical tape. All surgical procedures were carried out under a combination of nembutal and ether anaesthesia with aseptic precautions. A length of wide bore glass tubing was passed over the tail and lead, and connection by way of soldered joints made to a long cable. The rat was maintained in a polythene bucket and was effectively restrained by tying the cable at a point vertically overhead and by clamping the glass tubing so that it was firmly held over the tail. Under these conditions the animals were prevented from gnawing at the cable and yet remained in excellent health over periods of up to 15 days.

The $^{32}P$ was given by intraperitoneal injection of a solution of radioactive sodium phosphate in distilled water at the rate of 1 $\mu$Ci per 100 g. of body weight. This dosage gave a suitable counting rate. Drugs used for experimental purposes have all been given by intraperitoneal injection. Details of dosages are given in the appropriate section below.

### EXPERIMENTAL FINDINGS

After dosage with $^{32}P$ uptake has been rapid and a steady counting rate achieved after about 2 hours. Within 24–48 hours after insertion of the counter into the tumour and treatment with $^{32}P$ a regular pattern of counting has been achieved. This has normally been a steady rate showing only those irregularities to be expected as a result of the random decay of the $^{32}P$. A tracing of a normal recording is shown at Fig. 3*a*. The parallel lines denote the extremes of random variation of count-rate, being $\pm 3$ standard deviations from the local mean value. The age or sex of the host carrying the transplant appears to have had no effect upon either the count rate or the pattern of counting.

In a number of tracings significant spontaneous variations of count-rate have been seen. An example is shown in Fig. 3*b*, in which the parallel lines again

FIG. 3*a*.—Normal trace from osteosarcoma.
*b*.—Trace showing spontaneous peaking—fibrosarcoma.
Read from right to left.

denote the statistical limits of variation. Many regular variations of approximately 1½ hour period are present, superimposed on a large slower circadian rhythm. In other tracings rhythms of about 12 hour periods have been observed, both with and without faster components.

Numerous attempts have been made to induce rhythmic variations in the count rate as Bullen *et al.* (1963) have shown is possible with some human tumours. Methotrexate has been given in single or multiple doses at various levels ranging between 0·6 mg./kg. bdy.wt. and 10·0 mg./kg. bdy.wt. No example of any satisfactory response has been obtained.

Treatment with a number of physiologically important compounds has been undertaken to see if they influence the counting rate. Sucrose (10 g./kg.

bdy.wt.) insulin, vasopressin and corticosterone have had no effects on the counting rate. Somatotrophin, which might be expected to cause the tumours to grow faster does increase the counting rate for several days after a single dose of 1 i.u. There is a delay of 24 hours between treatment and the appearance of the enhanced count rate. Both tumours behave similarly.

Because of the clinical evidence of variations in radiosensitivity in accord with the occurrence of peaks and troughs in the count rate from a tumour tests of known radioprotective and radiosensitising agents have been made with this system.

Cysteine was shown by Patt, Tyree, Straube and Smith (1949) to be an effective radioprotector. It was given in neutralised solution at the maximum tolerated dose of 1 g./kg. bdy.wt. No immediate effect on the counting rate occurred in either tumour. In some but not all cases a slightly increased counting rate became apparent about 24 hours after treatment. This effect lasted for 3–4 days and then the count rate returned to a normal level.

Cysteamine ($\beta$-mercaptoethylamine) was first described as a radioprotective agent by Bacq, Herve, Lecomte, Fischer, Blavier, Deschamps, Le Bihan and Rayet (1951). It was given in buffered solution at pH 7·5 at a dosage of 125 mg./kg. bdy.wt. It caused a rapid and obviously statistically significant fall in the count rate from either tumour (Fig. 4). Recovery to a normal level was also rapid. The time of the maximum depression of counting was $\frac{3}{4}$–1 hour after treatment.

2 Aminoethylisothiouronium bromide (A.E.T.) was considered by Doherty and Burnett (1955) to be a more active protector than cysteamine. This was used at a dosage of 300 mg./kg. bdy.wt. and was found to induce similar effects to those obtained with cysteamine (Fig. 4*b*). Again the timing was similar to that with cysteamine.

2,4-Dinitrophenol, apart from its well known uncoupling action on oxidative phosphorylation, is also a radioprotective agent (Praslicka, Hill and Novak, 1962). This was used at the rate of 6 mg./kg. bdy.wt. and resulted in a rapid fall in the counting rate (Fig. 4*c*). Return to a normal level did not take place until 48 hours had elapsed.

Since three out of the four radioprotective agents used caused a reduction in the $^{32}$P counting rate, several claimed radiosensitisers were also tested to see if they increased the counting rate. The results in these tests have not been so clear cut.

Neither iodoacetic acid (40 mg./kg. bdy.wt.) found to be a radiosensitiser of mice by Langendorff and Koch (1954) nor p-chloromercuribenzoic acid (18 mg./kg. bdy.wt.) found to radiosensitise mice by Patt, Mayer and Smith (1952) had any influence on the count rate from either tumour. Stein and Griem (1958) found L-triiodothyronine to radiosensitise a rat chloroleukaemia. This was given in a single dose of 0·2 mg./kg. bdy.wt. and resulted in a slight rise in the count rate about $1\frac{1}{2}$ hours after treatment (Fig. 5*a*). Synkavite (tetrasodium-2-methyl-1-4-naphthohydroquinone diphosphate) was first introduced by Mitchell (1948). Since that time various conflicting reports as to its effectiveness have appeared (see Foye, 1963). It was used as a single dose at the rate of 100 mg./kg. bdy.wt. and caused a small increase in the counting rate for several hours after treatment with both tumours (Fig. 5*b*). This rise in counting rate could be obtained repeatedly. The mean levels were assessed over periods of 3 hours, before administration of Synkavite, and after the resulting rise had stabilised. Stating errors in terms of $\pm 2$ standard deviations (corresponding to a 95% confidence) the mean level before administration was $1 \cdot 17 \pm 0 \cdot 04$ c./sec., while the mean final level was $1 \cdot 36 \pm 0 \cdot 04$ c./sec. Thus, although the rise is only about 15%, it has high reality.

FIG. 4*a*.—Effect of 125 mg./kg. cysteamine—fibrosarcoma.
*b*.—Effect of 300 mg./kg. A.E.T.—osteosarcoma.
*c*.—Effect of 6 mg./kg. 2,4-dinitrophenol—osteosarcoma.
Read from right to left.

FIG. 5*a*.—Effect of 0·2 mg./kg. triiodothyronine—fibrosarcoma.
*b*.—Effect of 100 mg./kg. Synkavite—osteosarcoma.
Read from right to left.

The most frequently used method for the sensitisation of tumour cells to irradiation is to increase oxygenation. In some preliminary trials the rats have been maintained for periods up to 1 hour in oxygen at 1 atmosphere pressure. This has had no effects on the counting rate from either tumour.

### DISCUSSION

The results described above have been obtained with two tumours and one strain of rat. Under these circumstances too much value must not be placed on them. Nevertheless, certain points of interest have become apparent.

It has been clearly shown that the technique of counting of $^{32}P$ from tumours, originally developed for use with human patients, can be satisfactorily applied to transplanted tumours in small animals. The only specific requirement would appear to be the use of solid tumours which grow sufficiently large to allow a thickness of 8 to 10 mm. between the counter and any normal body structure. This is to avoid counting from normal tissues. This requirement has been met in the present experiments since both tumours grow to a considerable size without the occurrence of large masses of necrotic tissue.

Under normal circumstances the counting rate has followed an expected pattern of regularity consistent with the effective decay of the isotope. This fact in itself has made the system an excellent one for testing the effects of extraneous agents. Exceptionally, spontaneous fluctuations were encountered in the count rate with a timing of approximately two hours between peaks. A similar timing was found by Bleehen, Bryant and Gallear (1965) to occur between spontaneous peaking found in the count rate from hormone dependent carcinogen induced mammary tumours in Sprague-Dawley rats. Although this timing differs considerably from the 13 hour mean interval found between peaks in humans by Hale (1961) the occurrence of rhythms of about 12 hours period in some tracings suggests that the behaviour of $^{32}P$ in rat tumours runs parallel to that of $^{32}P$ in human tumours.

Of the four radioprotective agents used three caused a fall in the counting rate, a fact which appears consistent with the clinical experience that a trough in the counting rate is associated with a period of relative radio-insensitivity. Experience with 2,4-dinitrophenol is interesting since the counting rate was depressed over a long time. Although usually regarded as an acute poison, Dianzani and Scuso (1956) found that the uncoupling effect of 2,4-dinitrophenol on rat tissues persisted for more than 20 hours. This, however, was not confirmed by Parker (1956). The present results certainly indicate that an upset in phosphorus levels induced by 2,4-dinitrophenol can persist for a long period.

The fact that cysteine failed to have an immediate effect on the counting rate may or may not be significant. It is possible that the cysteine failed to reach the tumour. This explanation receives some support from the fact that in a limited series of experiments with the fibrosarcoma it was found that cysteine at a dosage of 1 g./kg. bdy.wt. failed to increase the non-protein sulphydryl level of the tumour up to 1 hour after treatment. During the same interval the non-protein sulphydryl levels of the liver and spleens of the same rats increased considerably. Alternatively, it is possible that the protection afforded by cysteine is mediated by a different mechanism from that afforded by cysteamine or A.E.T. Such a possibility has been suggested by Bacq (1965) and is further supported by consideration of the vastly greater (in terms of available thiol) amount of cysteine required to give protection as compared with other thiol protectors.

The increased counting rate found on occasion after a single dose of cysteine might be associated with an increased growth rate of the tumour. Haddow (1958) has shown that single doses of thiol compounds can induce an increased tumour growth rate.

Results obtained with the radiosensitisers show the interesting feature that both caused a small rise in counting rates. This would appear to be in agreement with the clinical findings. Failure of the other agents used to alter the $^{32}P$ counting rate does not signify that they are useless, it merely means that they have not altered the levels of $^{32}P$ in the currently used tumours.

The present work enables it to be said that the levels of phosphorus in experimental tumours are not necessarily constant, may show spontaneous rhythms and can be significantly altered by external agents. The alteration of phosphorus levels by agents altering radiosensitivity raises the new question of the role of intracellular phosphorus in tissue response to irradiation. Further work along the present lines would appear desirable as it seems likely to open new prospects in terms of radiation biochemistry and offer a basis for further experimental work aimed at elucidation of clinical findings.

SUMMARY

The physical background and instrumentation for the continuous counting of $^{32}$P from tumours has been described. The application of these methods in the case of two transplantable tumours in August rats is outlined. The effects of a number of physiologically important agents and some known radioprotective or radiosensitising agents on $^{32}$P counting rates have been examined. The findings are discussed.

We are indebted to Dr. R. C. Tudway and Dr. B. T. Hale for much helpful discussion; to D. Bussell, L. A. Daynes, A. S. Burleton and Miss S. Wheeler for technical assistance and to F. Butcher for his care of the animals.

The expenses of this work have been defrayed from a block grant to Mount Vernon Hospital from the British Empire Cancer Campaign for Research, and grants to Bristol General Hospital from the British Empire Cancer Campaign for Research, the Medical Research Council, the United Bristol Hospitals and the Damon Runyon Memorial Fund.

### REFERENCES

BACQ, Z. M.-(1965) ' Chemical Protection Against Ionising Radiation '. Springfield (C. C. Thomas), p. 247.

BACQ, Z. M., HERVE, A., LECOMTE, J., FISCHER, P., BLAVIER, J., DECHAMPS, G., LE BIHAN, H. AND RAYET, P.—(1951) *Archs int. Physiol.*, **59**, 442.

BLEEHEN, N. M., BRYANT, T. H. AND GALLEAR, R.—(1965) 'Abstrs. 11th Int. Congress of Radiol ' p. 464.

BULLEN, M. A., FREUNDLICH, H. F., HALE, B. T., MARSHALL, D. H. AND TUDWAY, R. C. —(1963) *Post-grad. med. J.*, **39**, 265.

DIANZANI, M. U. AND SCUSO, S.—(1956) *Biochem. J.*, **62**, 205.

DOHERTY, D. G. AND BURNETT, W. T.—(1955) *Proc. Soc. exp. Biol. Med.*, **89**, 312.

FOYE, L. V.—(1963) ' The Modification of Radiosensitivity, in Cancer '—Progress Volume 1963, London (Butterworths) p. 208.

HADDOW, A.—(1958) *Rep. Br. Emp. Cancer Campn*, **36**, 77.

HALE, B. T.—(1961) *Lancet*, ii, 345.

LANGENDORFF, R. AND KOCH, R.—(1954) *Strahlentherapie*, **95**, 535.

MITCHELL, J. S.—(1948) *Br. J. Cancer*, **2**, 351.

PARKER, V. H.—(1956) *Nature, Lond.*, **178**, 261.

PATT, H. M., MAYER, S. H. AND SMITH, D. E.—(1952) *Fedn Proc. Fedn Am. Socs exp. Biol.*, **11**, 118.

PATT, H. M., TYREE, E. B., STRAUBE, R. L. AND SMITH, D. E.—(1949) *Science, N.Y.*, **110**, 213.

PRASLICKA, M., HILL, M. AND NOVACK, L.—(1962) *Int. J. Radiat. Biol.*, **4**, 567.

STEIN, J. A. AND GRIEM, M. L.—(1958) *Nature, Lond.*, **182**, 1681.